# Macrophage activation syndrome (MAS) in different pediatric rheumatic disease

**DOI:** 10.1186/1546-0096-12-S1-P220

**Published:** 2014-09-17

**Authors:** Zubeyde Gunduz, Betul Sozeri, Ruhan Dusunsel, Hakan Poyrazoglu, Ismail Dursun, Aysenur Pac Kisaarslan, Sibel Yel, Kenan Yilmaz

**Affiliations:** 1 Pediatric Rheumatology, Erciyes University, Kayseri, Turkey; 2 Pediatric Rheumatology, Ege University, Izmir, Turkey

## Introduction

Macrophage activation syndrome (MAS) is a life-threatening complication of chronic rheumatic disease in childhood.

## Objectives

We aim to eveluate MAS findings and outcomes that differ according to disease in childhood.

## Methods

We obtain 11 rheumatic patients followed in two different pediatric rheumatology units (Erciyes University and Ege University) who presented with MAS. We report their clinical and laboratory findings, therapies and outcomes.

## Results

The primary diagnoses of the patients included in the study, respectively; systemic juvenil idiopathic arthritis (n=5), Systemic Lupus Erythematosus (n=2), juvenile dermatomyositis (n=2), a neonatale onset multisystem inflammatory disease (NOMID) and a microscopic polyartritis nodosa. The mean age of the patients was 9.9 years old (1-14), and male to female ratio was 3:8. The mean duration of underlying disease was 6 months (1-24 months) at the diagnosis of MAS. We found MAS due to infection in four patients ,while used medicine in a patient. MAS were developed spontaneously in 6 patients. The clinical manifestations of MAS included fever 7 (63.6%),mucosal bleading 6 (54.5%), neurologic involvement 4 (36.4%) and hepatomegaly 6 (54.5%).

We found thrombocytopenia in 9 (81.8%), leucopenia in 5 (45.5 %), increased AST in 7 (63.6%), hypofibrinogenemia in 6 (54.5%), increased ferritin in 11 (100%), decreased ESH in 4 (36.4%) and increased triglyceride in 10 (90.9%) patients. We investigated bone marrow in all patients, and hemophagocytosis were determinated in 8 (72.7%). The medications were pulse methylprednisolone 6 (54.5%), intravenous immunoglobulin 8 (72.7%), plasma exchange 5 (45.5%), cyclosporine 6 (54.5%), dexamethasone 1 (9.1%), etoposide 1 (9.1%). The prognosis of patients were recovery 8 (72.7%), and exitus 3 (27.3%).

## Conclusion

In conclusion, MAS can be developed in various pediatric rheumatologic disease and fatal. Prompt recognition and timely treatment can result good outcomes.

## Disclosure of interest

None declared.

